# Reconstruction of the treatment area by use of sinogram in helical tomotherapy

**DOI:** 10.1186/s13014-014-0252-0

**Published:** 2014-11-28

**Authors:** Akihiro Haga, Keiichi Nakagawa, Calvin Maurer, Ken Ruchala, Edward Chao, Dylan Casey, Satoshi Kida, Dousatsu Sakata, Masahiro Nakano, Taiki Magome, Yoshitaka Masutani

**Affiliations:** Department of Radiology, University of Tokyo Hospital, 7-3-1 Hongo, Bunkyo, Tokyo Japan; Accuray Incorporated, Sunnyvale, CA USA

**Keywords:** Megavoltage CT, In-treatment CT, Image reconstruction during treatment, TomoTherapy®

## Abstract

**Background:**

TomoTherapy (Accuray, USA) has an image-guided radiotherapy system with a megavoltage (MV) X-ray source and an on-board imaging device. This system allows one to acquire the delivery sinogram during the actual treatment, which partly includes information from the irradiated object. In this study, we try to develop image reconstruction during treatment with helical tomotherapy.

**Findings:**

Sinogram data were acquired during helical tomotherapy delivery using an arc-shaped detector array that consists of 576 xenon-gas filled detector cells. In preprocessing, these were normalized with full air-scan data. A software program was developed that reconstructs 3D images during treatment with corrections as; (1) the regions outside the field were masked not to be added in the backprojection (a masking correction), and (2) each voxel of the reconstructed image was divided by the number of the beamlets passing through its voxel (a ray-passing correction).

The masking correction produced a reconstructed image, however, it contained streak artifacts. The ray-passing correction reduced this artifact. Although the SNR (the ratio of mean to standard deviation in a homogeneous region) and the contrast of the reconstructed image were slightly improved with the ray-passing correction, use of only the masking correction was sufficient for the visualization purpose.

**Conclusions:**

The visualization of the treatment area was feasible by using the sinogram in helical tomotherapy. This proposed method would be useful in the treatment verification.

## Introduction

As radiotherapy is complex, treatment verification becomes significant. The evaluation of the absorbed dose in phantoms is strongly recommended for all patients having intensity-modulated radiotherapy (IMRT) [1]. In addition, accuracy of the patient setup is more important in the IMRT than that in the conventional radiotherapy. Image-guided radiotherapy (IGRT) can entail correcting the patient position just prior to treatment by gathering information about anatomical locations during setup. IGRT can utilize various imaging technologies such as the portal images of the treatment beam [2-5], magnetic resonance imaging [6], ultrasound [7,8], and computed tomography (CT) [9,10].

TomoTherapy® has an IGRT system with megavoltage (MV) X-ray source and an on-board imaging device [11]. With the MV CT, it became feasible to perform efficient daily-3D registration of the patient position before each treatment delivery. This system also allows one to acquire the delivery sinogram during the actual treatment. The sinogram has often been used in the treatment verification [12,13], and one can come up with the visualization of treatment area from the sinogram. For conventional linear accelerators, in fact, CT reconstruction with portal images during rotational treatment such as a volumetric modulated arc therapy (VMAT) has been successfully performed [14,15]. Also the MV CT reconstruction during treatment in helical TomoTherapy® delivery has been first tried in Ref. [16], where the insertion of full field-of-view (FOV) beamlets was cooperated with the treatment sinogram. In this note, we focus on the image reconstruction of treatment area without full FOV insertion.

For the preliminary arrangement, we developed a helical CT reconstruction algorithm that includes corrections for the heterogeneous beam profile and the geometrical disagreement between the X-ray source position and the detector curvature. Then, the feasibility of the reconstruction of the treatment area was examined with the treatment sinogram to a phantom, which includes the information of the irradiated part of the phantom.

## Materials and methods

**Figure 1:**
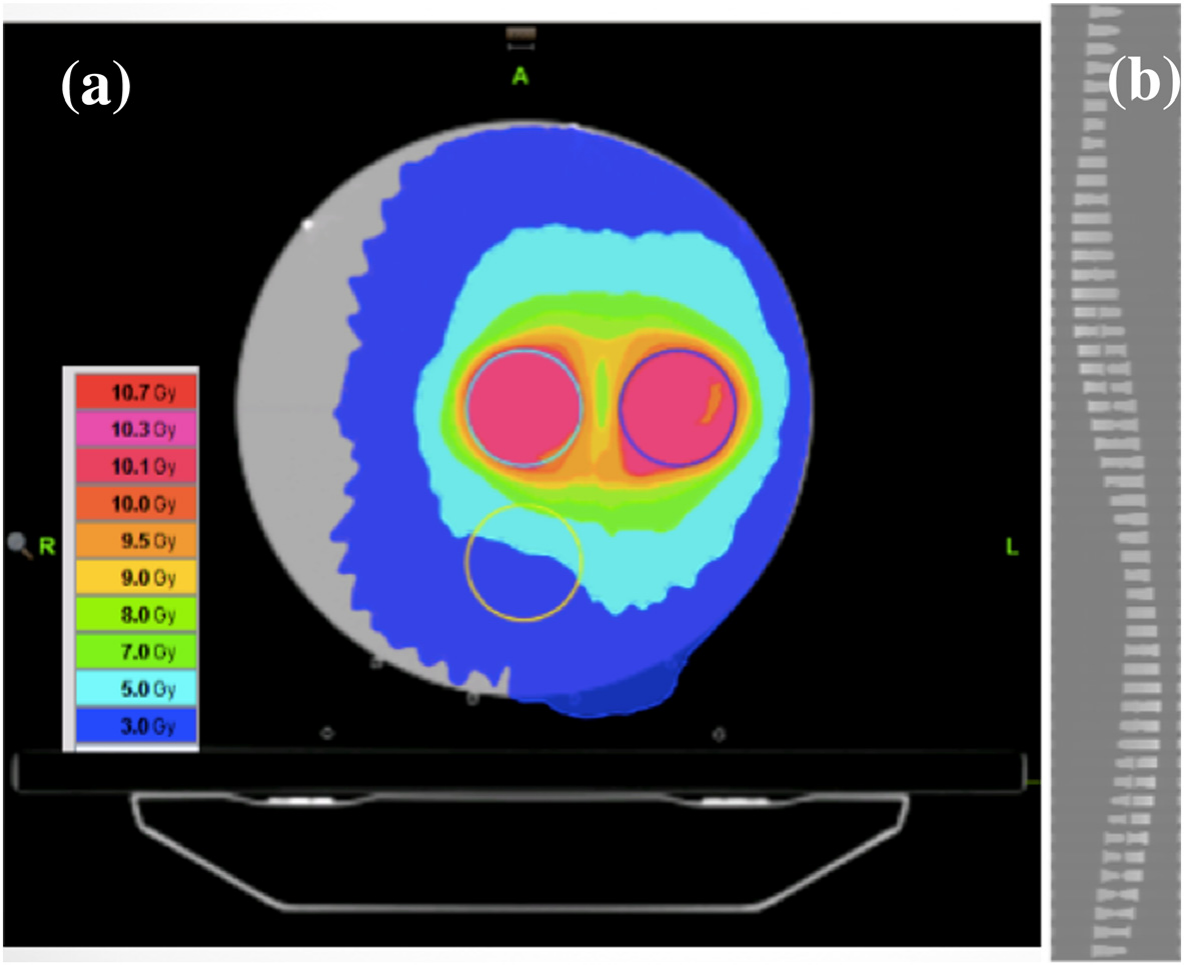
**(a) Dose distribution of the factory IMRT test plan in the TomoPhantom® and (b) corresponding delivery sinogram.**

All data were obtained on a TomoTherapy® unit at Accuray in Madison, Wisconsin. The factory IMRT test plan was irradiated to TomoPhantom® (Accuray, USA) with density plugs. This test plan prescribes 10 Gy for two cylindrical targets (3 cm radius and 6 cm length) which are seen as blue and sky-blue circles in Figure 1(a). Sinogram data were acquired during helical TomoTherapy® deliveries using an arc-shaped detector array consisting of 576 xenon-gas filled detector cells, of which the data from middle 527 cells were used in the reconstruction. The data has the following acquisition properties: gantry rotation period 14 s, data sampling rate 300 Hz, starting view angle 229.371°, and couch travel distance over entire data sample 90.6 mm. There has 52,788 samples, and thus, the couch speed was 0.515 mm/s.

The dose distribution in the treatment planning system (TPS) and a part of the corresponding delivery sinogram are shown in Figures 1(a) and (b), respectively. The sinogram was normalized with full air-scan data to the correct heterogeneous beam profile and modulated beam intensity of TomoTherapy®.

The reconstruction was performed with an in-house program employing the filtered back projection (FBP) algorithm using Shepp-Logan filter. Because the source-to-isocenter distance (85.0 cm) is smaller than the detector radius of curvature (99.8 cm), our program converts the original data in each detector cell into the virtual one with the curvature corresponding source-to-isocenter geometry. Then, the virtual data with a constant cell-to-cell interval was created by linear interpolation.

In general, it is impossible to make a *correct* reconstruction with a limited FOV using FBP [14]. As shown in Figure 1(b), the area blocked by binary multileaf collimator (MLC) in the sinogram has a lower X-ray intensity than that inside the FOV, so that the conventional reconstruction scheme does not successfully visualize the object. Instead this yields an unrealistically high-attenuation area outside the irradiated site in the object. For the visualization of the irradiated area, therefore, we employed two corrections. One was a masking correction, which masks the area outside the FOV so as not to include this area in the backprojection process. For this, sinogram normalized by full air scan, $\tilde{P}_{\beta}\left(\gamma\right)$ where *β* and *γ* denote the gantry and fan angles, respectively, can be expressed as,

1 $$\tilde{P}_{\beta }\left(\gamma\right)=\kern1.5em \left\{\begin{array}{l}1, \kern7.75em  if\ {P}_{\beta}\left(\gamma \right)/{P}_{\beta}^{air}\left(\gamma \right)<{p}^{\hbox{'}}\hfill \\ {}{P}_{\beta}\left(\gamma \right)/{P}_{\beta}^{air}\left(\gamma \right), \kern4.25em  otherwise\hfill \end{array}\right. $$

where *p*^’^ means the threshold for masking region, and here, we employed *p*^’^ = 0.2. With this correction, the outside field is regarded as air and the boundary of masking region is discontinuous. Of course, this is not true, but it enhances the information from the irradiated area in the FBP reconstruction scheme.

The other correction was a ray-passing adjustment, which normalized each voxel of the reconstructed image to the number of the X-rays passing through the corresponding voxel. Namely, using the masking function,

2 $$ M\left(\gamma, \beta \right) = \left\{\begin{array}{l}\kern1em 1, \kern2.25em  if\ {p}^{\hbox{'}} < \tilde{P}_{\beta}\left(\gamma \right)<1\hfill \\ {}\kern1em 0, \kern6em  otherwise\hfill \end{array}\right., $$

the correction factor for ray-passing can be expressed as,

3 $$ R\left(\gamma, {\beta}^{\hbox{'}}\right)=\frac{\Theta \left({\displaystyle {\int}_0^{2\pi }}M\left(\gamma, \beta \right)d\beta -{\beta}^{\hbox{'}}\right)}{{\displaystyle {\int}_0^{2\pi }}M\left(\gamma, \beta \right)d\beta }\ . $$

The backprojection generates stronger signals from the angles passing more X-rays. The ray-passing correction corrects this effect. The reconstructed region was controlled by *β*^’^ in Eq. (3). In this study, the area irradiated with more than 35% (*R*(*γ*, *β*^’^) = 0.35) and 55% (*R*(*γ*, *β*^’^) = 0.55) of the maximum was reconstructed and the other area was masked.

The contrast in the images was evaluated by the ratio of the signal in high-density regions to that in low-density regions in the object. The homogeneity was evaluated from the three regions that are composed of the same material. The signal-to-noise ratio (SNR) inside the region-of-interest (ROI) was also evaluated.

## Results and discussion

**Figure 2:**
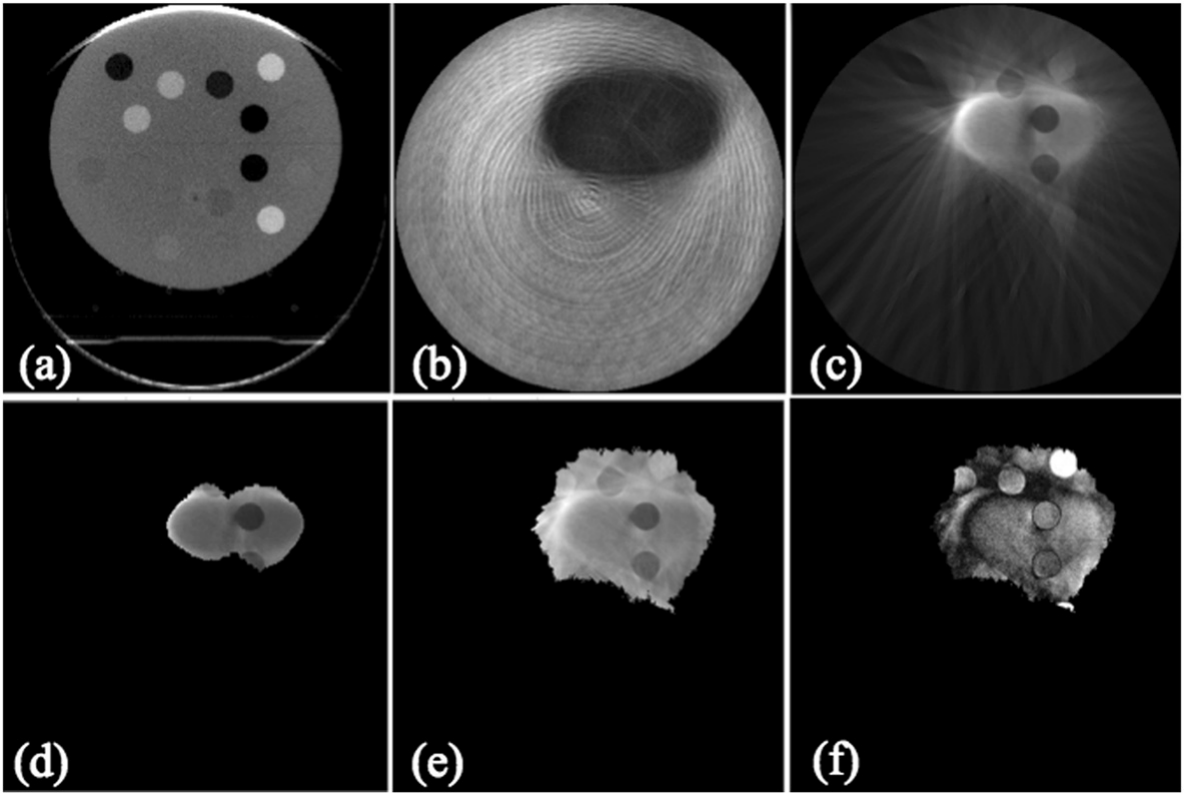
**Reconstruction images; (a) with full FOV, (b) with no masking and no ray-passing correction, (c) with masking correction only** **(** ***p*** ^’^ **= 0.2),** **(d) with both corrections and** **(** ***p*** ^**'**^ **= 0.2 and** ***R*** **(** ***γ*** **,** ***β*** ^**'**^ **) = 0.55)**, **(e) with both corrections with broader reconstruction and** **(** ***p*** ^**'**^ **= 0.2 and** ***R*** **(** ***γ,*** ***β*** ^**'**^ **) = 0.35),** **and (f) the overlapped image between (a) and (e).** The image **(a)** was reconstructed with 4 mm/rotation, whereas the others were reconstructed with 7.2 mm/rotation which can depend on the treatment plan.

Figures 2(a)-(f) show the reconstructed images using a full FOV, with no masking and no ray-passing corrections, with masking correction only, and with both corrections, respectively. Without masking and ray-passing corrections (Figure 2(b)), the visualization of the treatment area was poor. Use of the masking correction and the ray-passing correction improved the reconstructed image for the treatment site (Figures 2(c)-(e)). The masking correction made the image clearly visible, however, a streak artifact was introduced (Figure 2(c)). The ray-passing correction reduced this artifact (Figures 2(d) and (e)). In Figure 2(f), the image difference between Figures 2(a) and (e) is shown. It is found that the location and the size of inner plugs were well reproduced in Figure 2(f). On the other hand, the difference can depend on the density plug, because of the low contrast of Figure 2(e) in comparison with Figure 2(a).

**Figure 3:**
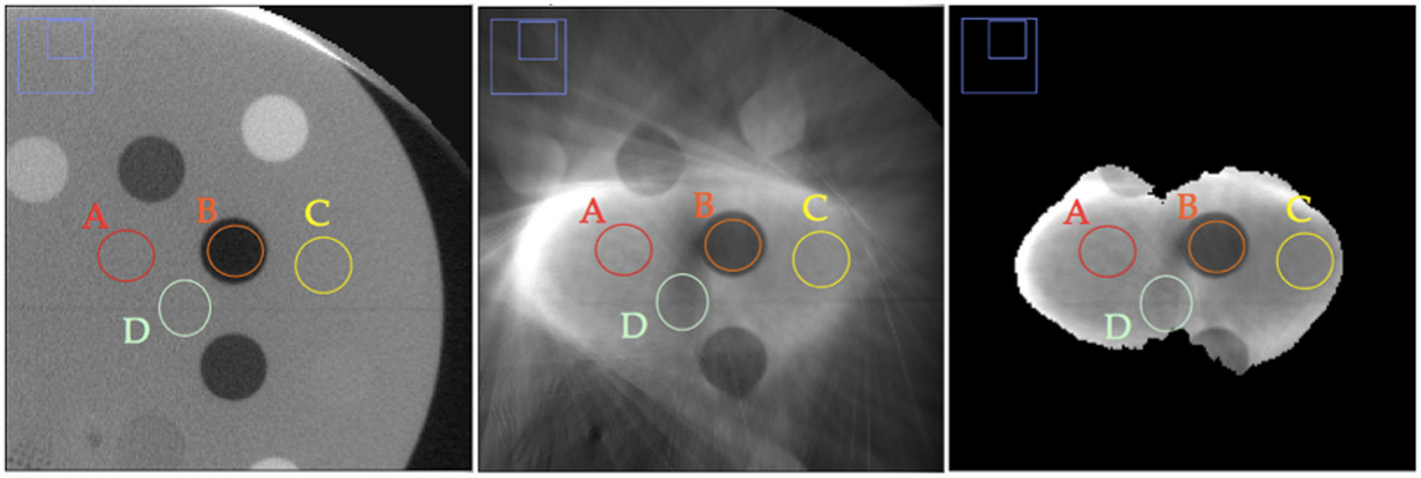
**Enlarged displays of the reconstruction images with full FOV (left panel), with masking correction only (middle panel), and with both corrections (right panel).**

Table 1 shows the quantitative evaluation of contrast, homogeneity, and SNR. The contrast index was calculated by the ratio of the mean value of regions A, C, and D to that of region B (see Figure 3). As expected, the contrast for the in-treatment images is considerably poorer than for the full FOV image. The homogeneity, as evaluated by the *min-max* values of the mean value in the regions A, C, and D has the same tendency. On the other hand, the SNR can be enhanced in the in-treatment images, presumably, due to the blurring effect.

**Table 1:** **Results of contrast, homogeneity, and SNR analyses**

**Reconstruction**	**Contrast**	**Homogeneity**	**SNR @ A**	**SNR @ B**	**SNR @ C**	**SNR @ D**
Full FOV	6.17	0.99-1.02	19.61	2.98	16.97	19.91
Masking only	1.79	0.83-1.15	18.13	16.28	9.33	55.50
Full corrections	1.89	0.91-1.06	20.47	16.70	10.53	44.44

The contrast was evaluated by the ratio of the mean value of regions A, C, and D to that of region B in Figure 3. The homogeneity was evaluated by the *min-max* values of the mean value in the regions A, C, and D.

In the analysis of image contrast and homogeneity, the ray-passing correction improved the image quality, but no dramatic change in visibility was yielded. Of course, this is not a general conclusion. One set of questions might be to further examine how well this type of technique works for different cases, such as different target sizes, different anatomical regions, and with different levels of leaf modulation.

Although a further study will be required, the present result encouraged us to develop the record-and-verify system with the reconstructed delivery area from the actual treatment. Although a further study will be required, the present result encouraged us to develop the record-and-verify system with the reconstructed delivery area from the actual treatment. Also one may be interested in the dose reconstruction using present method. The present method cannot be applied for the dose reconstruction directly. However, the dose reconstruction in each treatment session requires the information of the patient location during treatment, which can be provided by the present method. Thus, the development of the image reconstruction using the delivery sinogram would be a promising tool for in-vivo dosimetry as well as for verification of irradiated areas.

## Conclusion

A reconstruction technique using the treatment sinogram has been developed for helical Tomotherapy. The improved visibility of structures in the reconstructed image makes this a promising tool for verifying relative anatomical positions during the course of a treatment.
